# Diagnostic center for primary care patients with nonspecific symptoms and suspected cancer: compliance to workflow and accuracy of tests and examinations

**DOI:** 10.1080/02813432.2021.1913892

**Published:** 2021-05-06

**Authors:** Emelie Stenman, Karolina Palmér, Stefan Rydén, Charlotta Sävblom, Jianguang Ji, Jan Sundquist

**Affiliations:** a Center for Primary Health Care Research, Department of Clinical Sciences Malmö, Lund University, Sweden; b Regional Cancer Centre South, Skåne Regional Council, Lund, Sweden; c Regional Cancer Centre Stockholm Gotland, Stockholm Regional Council, Stockholm, Sweden; d Department of Family Medicine and Community Health, Icahn School of Medicine at Mount Sinai, New York, NY, USA; e Department of Population Health Science and Policy, Icahn School of Medicine at Mount Sinai, New York, NY, USA; f Department of Functional Pathology, School of Medicine, Shimane University Japan, Center for Community-based Healthcare Research and Education (CoHRE), Matsue, Japan

**Keywords:** Neoplasms, cancer, nonspecific symptoms, guideline adherence, test accuracy

## Abstract

**Objective:**

To evaluate compliance to workflow and accuracy of tests in Sweden’s first fast-track referral pathway for patients with nonspecific symptoms and suspected cancer (SCAN).

**Design:**

Prospective cohort study with consecutive inclusion of patients referred to the diagnostic center (DC).

**Setting:**

Patients with nonspecific symptoms were examined in primary care according to a protocol including two test packages and diagnostic imaging. If symptoms were not explained, patients were referred to the DC and a DC-test package was taken. At the DC, further investigations resulted in diagnosis/no diagnosis.

**Subjects:**

A total of 290 patients, median age 69 years (interquartile range [IQR] 59–76), 48% men, participated. A total of 64 (22%) were diagnosed with cancer, 186 (64%) with non-malignant disease and 40 (14%) had no new disease.

**Main outcome measure:**

Compliance was estimated by percentage of compulsory tests taken. Test accuracy was assessed by likelihood ratios (LRs) regarding cancer.

**Results:**

A total of 23 (8%) patients had taken both primary care packages, whereas 150 (52%) patients went through entire diagnostic imaging. Abnormal pulmonary X-ray, peak expiratory flow (PEF) and calcium had the highest LRs in primary care (3.5; 3.2; 2.7). A total of 105 (36%) took the complete DC-package, of which bilirubin and cytomegalovirus had the highest LRs (11.5; 10.9). The median number (IQR) of abnormal primary care tests was 5 (3–6) for cancer, 3 (2–6) for other diagnoses and 1 (0–3) for no diagnosis.

**Conclusions:**

Compliance to test packages in primary care was low, which warrants review of the workflow. Few single tests had high accuracy regarding cancer, but the number of abnormal tests can provide guidance in complicated investigations of suspected malignancies.

**KEY POINTS**
- Fast-track referral pathways for patients with nonspecific serious symptoms have been implemented in several countries and are part of the national cancer strategy in all of Scandinavia.
- Compliance with compulsory tests in primary care was modest in this study; 8% of the patients had taken the entire compulsory test packages.
- Few single compulsory tests had high accuracy regarding subsequent cancer, which warrants a review of tests and examinations. However, patients diagnosed with cancer had a higher number of abnormal test results compared to the other groups.

## Introduction

Previous research regarding length of diagnostic intervals *versus* clinical outcome for cancer has resulted in different conclusions, from positive to negative or u-shaped associations [1–6]. However, it is a fact that most malignant tumors progress with time and that more advanced tumor stage is associated with worse prognosis [7,8], thus, timely cancer diagnoses should, in general, be targeted. Patients with nonspecific symptoms may be disadvantaged in this regard due to symptoms that may take longer time to be recognized as signs of serious disease [9], and also due to more complicated and prolonged investigations compared to patients with early alarm symptoms. This was confirmed in a study of six common cancer forms, which showed longer diagnostic intervals and more advanced stage tumors for patients with vague symptoms [6]. Studies have shown that less than half of patients later diagnosed with cancer displayed alarm symptoms initially [10–12].

Fast-track referral pathways for patients with nonspecific, serious symptoms have been implemented nationwide in Sweden and all Scandinavia as part of the national cancer strategy [13–15]. The original concept ‘Nonspecific symptoms and signs of cancer patient pathway’ (NSSC-CPP) stems from Denmark [15–17] and a similar pathway, Suspected CANcer (SCAN), has been introduced in England [18].

Sweden’s first diagnostic center (DC) for nonspecific symptoms, which opened in 2012, is currently being evaluated. A previous study examined the diagnostic spectrum, time intervals and patient satisfaction and showed that the average investigational time at the DC met the expected goal of 22 days. However, in primary care, the time goal was not achieved; less than half of the patients were investigated within the expected 15 days. It turned out that patients who did not fulfill the time goals had a higher degree of incomplete investigations [13]. The main purpose of this study was to evaluate compliance to the workflow and accuracy of recommended tests and examinations in the first Swedish DC model. Regarding compliance, comparisons were made between patients diagnosed with cancer, diagnosed with other, non-malignant, diseases and those who did not get a diagnosis. Accuracy was estimated by analyzing sensitivity, specificity, positive and negative predictive values, likelihood ratios (LRs) and post-test probabilities of tests and examinations to see which contributed most to cancer detection.

## Material and methods

Sweden’s first DC was established at the Kristianstad Central hospital as a separate, outpatient unit within the Department of internal medicine. The catchment area was comprised of 42 primary healthcare centers and 220,000 inhabitants. A project group developed, together with representatives from primary care, a communication plan and implementation commenced in 2011 with information meetings and distribution of written information to all healthcare centers before the DC opened in October 2012. The project group continued to visit the healthcare centers throughout the study to ensure awareness of the DC model and get routines working.

Much of the design was adopted from the Danish model. The workflow and methods of the evaluation have been described previously [13]. In short, primary care physicians were invited to refer patients 18 years or older with one or more of the following symptoms: (1) fatigue, (2) weight loss more than 5 kg, (3) pain/joint pain, (4) prolonged fever, (5) abnormal lab values or (6) suspected metastasis with a lack of focal cancer symptoms [15,19]. Patients referred to the DC were consecutively invited to participate in the study, except for those who were unable to provide an informed consent based on oral and written study information.

### Investigation in primary care

**Figure 1.**
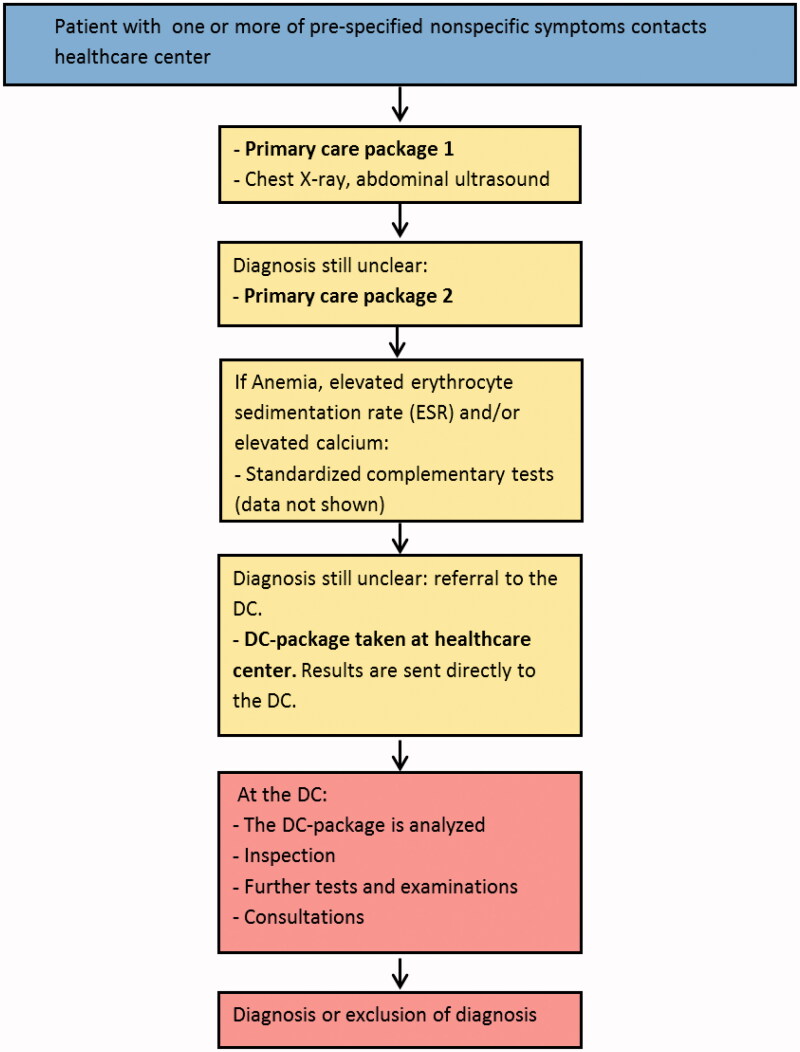
The DC workflow. Contents of primary care packages 1 and 2 and the DC-package are shown in Table 1.

Individuals who contacted their healthcare centers and met the inclusion criteria were offered an appointment with a physician within three working days. The diagnostic workup included a medical history, a clinical examination and two standardized sets of laboratory tests (Primary care packages 1 and 2, Table 1). Diagnostic workup also included pulmonary X-ray and abdominal ultrasound. Thereafter, if no explanation for the symptoms was found, patients were eligible for DC referral. In conjunction with referral, another panel of laboratory tests (DC-package, Table 1) was taken; the results being sent directly to the DC (Figure 1), thus not evaluated by the primary care physician.

**Table 1.** Test packages in the DC workflow.

Primary care package 1	Primary care package 2	DC-package
Hemoglobin (Hb) Erythrocyte sedimentation rate (ESR) C-reactive protein (CRP) Glucose Uric acid Feces hemoglobin Electrocardiography (ECG) Peak expiratory flow (PEF) Oxygen saturation	Thyroid-stimulating hormone (TSH) Blood leukocytes Creatinine Potassium Alanine aminotransferase (ALAT) Gamma-glutamyltransferase (GT) Alkaline phosphatase (ALP) Amylase Calcium Albumin Homocysteine	Iron TIBC Sodium Cystatin Calcium ion Uric acid Bilirubin NT-pro-BNP HbA1c a-tTG IgA ANA, anti-DNA, anti-ENA, anti-cardiolipin Hepatitis A Blood screening (HIV, hepatitis B and hepatitis C) Borrelia Epstein Barr virus Cytomegalovirus PSA (men)

ANA: antinuclear antibodies, a-tTG: anti-tissue transglutaminase, ENA: extractable nuclear antigens NT-pro-BNP: N-terminal pro-brain natriuretic peptide, PSA: prostate-specific antigen, TIBC: total iron-binding capacity.

### Investigation at the DC

Patients were offered an appointment with the DC-physician (specialized in internal medicine and family medicine) within three working days from referral. The diagnostic workup at the DC included the medical history and results from the DC package, a thorough physical examination (inspection) according to protocol and further appropriate tests, examinations and consultations. The diagnostic workup could result in a diagnosis or exclusion of diagnosis (Figure 1).

### Data collection

Data regarding symptoms, test results, investigations, comorbidity, drugs and diagnoses were collected by the DC’s physician and nurse in case report forms, which were monitored and validated by a study nurse. The results of tests and investigations were registered as ‘abnormal’ or ‘normal’ based on reference values (samples) or specialists’ judgement (physical examination and imaging).

### Statistical analysis

Patient characteristics were presented with median and interquartile range (IQR) (25th–75th percentile) or percentages. Compliance was estimated by the median number of tests taken in each group and number and percentage of patients that had taken the different tests. Test accuracy was assessed by sensitivity, specificity, positive and negative predictive values, LRs and negative and positive post-test probabilities. The LR was calculated as sensitivity/(1-specificity) and is an estimation of how much an abnormal test result increases the probability of cancer. Pre-test probability of cancer was equal to the actual cancer prevalence in the study sample. Post-test probabilities were estimated by predictive values after fitted logistic regression models and represent the probability of cancer after a normal test result (negative post-test probability), or after an abnormal test result (positive post-test probability). Confidence intervals of post-test probabilities were calculated by normal approximation with standard errors estimated using the delta method. All statistical analyses were done in STATA version 15.1 (StataCorp LP, College Station, TX).

## Results

### Study participants

Recruitment and characteristics of the study participants have been published elsewhere [13]. Between October 2012 and September 2015, at total of 499 patients were referred to the DC, of whom 393 were considered eligible for fast-track investigation. We do not have exact reasons for not inviting the remaining 106 patients, but some documented reasons were that the patients were not in shape for an out-patient unit investigation, that the patients denied investigation themselves, that the patients did not fulfill the referral criteria or that the patients did not belong to the catchment area. A total of 290 patients consented to take part in the scientific evaluation. 64 (22%) patients were diagnosed with cancer at the DC, 186 (64%) were diagnosed with non-malignant diseases, and 40 (14%) did not get a diagnosis. Table 2 shows the basic patient characteristics for each group. Patients diagnosed with cancer or other diseases were slightly older than patients without a diagnosis. The majority of patients diagnosed with cancer were men. Other diseases and no diagnosis were slightly more common among women. Among patients that did not get a diagnosis, a higher proportion had high education (>12 years) compared to the other groups. The share of patients that were born outside Sweden was 5%, which is low compared to the general Swedish population with a share of about 20%, suggesting a relatively homogenous group regarding country of birth.

**Table 2.** Patient characteristics and compliance to compulsory tests in primary care.

	All patients (*n* = 290)	Patients diagnosed with cancer (*n* = 64)	Patients diagnosed with other diseases, not cancer (*n* = 186)	Patients with no diagnosis (*n* = 40)
Age, median (IQR)	69 (59–76)	71 (65–77)	69 (59–76)	66 (53–74)
Sex (male/female)^a^, %	48/52	58/42	47/53	41/59
Education (low/middle/high)^a^, %	46/36/18	43/41/16	47/36/17	47/29/24
Marital status (married or live together/single)^a^, %	69/31	67/33	70/30	65/35
Born in Sweden (yes/no)^a^, %	95/5	94/6	94/6	97/3
Primary care package 1				
All 9 tests, number of patients	30 (10%)	2 (3%)	23 (12%)	5 (13%)
At least one test, number of patients	282 (97%)	61 (95%)	182 (98%)	39 (98%)
Number tests taken, median (IQR)	5 (4–7)	4 (3–5)	5 (4–8)	6 (4–7)
Primary care package 2				
All 11 tests, number of patients	89 (31%)	13 (20%)	61 (33%)	15 (38%)
At least one test, number of patients	282 (97%)	60 (94%)	183 (98%)	39 (98%)
Number tests taken, median (IQR)	9 (7–11)	8 (6–10)	9 (7–11)	10 (8–11)
Pulmonary X-ray, number of patients	191 (66%)	41 (64%)	125 (67%)	25 (63%)
Ultrasound, number of patients	165 (57%)	32 (50%)	109 (59%)	24 (60%)
Tests before referral to the DC^b^				
Number tests taken^c^, median (IQR)	15 (12–19)	14 (11–16)	15 (12–19)	17 (14–20)
Number abnormal, median (IQR)	3 (2–6)	5 (3–6)	3 (2–6)	1 (0–3)
Number normal, median (IQR)	11 (8–15)	8 (6–11)	12 (9–15)	15 (10–17)

^a^48 patients (17%) had missing information on any of sex, education, marital status or born in Sweden (23%, 14% and 18% in patients with cancer, other diseases and no diagnosis, respectively).

^b^Package 1 (9 possible tests), package 2 (11 possible tests) and pulmonary X-ray + ultrasound (in total 22 possible tests and examinations).

^c^Five patients (1.7%) did not take any test at all in primary health care (3, 2 and 0 in patients with cancer, other diseases and no diagnosis, respectively).

The symptom spectrum at referral to the DC has been described previously [13]. In short, the most common reason for referral was unexplained abnormal test results (57%) followed by weight loss (41%), fatigue (34%) and pain/joint pain (33%). Suspected metastasis (10%) and prolonged fever (3%) were less common. Patients diagnosed with cancer had a higher number of symptoms compared to patients with other diseases or no diagnosis. The most common cancer forms were hematological cancers, followed by lung cancer, colorectal cancer and secondary cancers [13].

### Compliance to test packages

Out of 22 compulsory tests and examinations in primary care, the median number per patient was 15 (IQR 12–19). Patients later diagnosed with cancer had a slightly lower number of compulsory tests taken (median 14, IQR 11–16). Fewer patients diagnosed with cancer took the complete test packages 1 and 2 compared to the other groups; the complete Primary care package 1 was taken in 30 (10%) of all patients, but only in 2 (3%) of patients later diagnosed with cancer and primary care package 2 showed a similar pattern. The vast majority of patients had taken at least one test in each package (Table 2). Only 23 (8%) patients had taken all the compulsory tests in primary care packages 1 and 2 before referral to the DC (data not shown), but a higher proportion of the patients were examined *via* diagnostic imaging. A total of 191 (66%) patients went through pulmonary X-ray and 165 (57%) abdominal ultrasound before DC referral (Table 2). A total of 150 (52%) patients went through both these examinations. Of the compulsory tests in primary care, hemoglobin (Hb), erythrocyte sedimentation rate (ESR), C-reactive protein (CRP), blood leukocytes, creatinine, potassium, Alanine aminotransferase (ALAT), Gamma-glutamyltransferase (GT) and Alkaline phosphatase (ALP) were taken in more than 80% of the patients. The least common tests were peak expiratory flow (PEF) and oxygen saturation which were measured in 18% and 24%, respectively (Supplementary Table 1).

As shown previously, 105 (36%) patients had taken the complete DC package in conjunction with referral to the DC and 235 (81%) patients had taken at least one of the tests [13]. The median number of tests taken in the DC package was 4 out of the 16 possible tests for women (or the 17 possible tests for men; data not shown). The only tests in the DC package that were taken in more than 60% of the patients were sodium and P-PSA (only men), but each test was taken in at least 37% of the patients (Table 3).

**Table 3.** Compliance to and test accuracy of the DC package.

Test	Taken *n* (%)	Abnormal *n* (%)	Normal *n* (%)	Sens %	Spec %	PPV %	NPV %	LR^a^	Negative post-test probability^b^ (95% CI)	Positive post-test probability^c^ (95% CI)
DC-package:										
Iron	165 (57)	51 (31)	114 (69)	48	73	28	87	1.8	16.7 (10.6–23.6)	33.3 (21.8–45.6)
IBC	163 (56)	16 (10)	147 (90)	14	91	25	83	1.5	21.0 (15.9–26.5)	30.8 (11.8–55.5)
Sodium	192 (66)	13(7)	179 (93)	16	96	46	82	3.5	19.9 (15.1–25.2)	49.7 (25.4–73.7)
Cystatin	121 (42)	30 (25)	91 (75)	41	79	30	86	1.9	17.4 (11.3–24.2)	35.2 (20.7–51.0)
Calcium ion	146 (50)	11 (8)	135 (93)	15	94	36	83	2.5	20.3 (15.2–25.7)	41.8 (16.5–69.0)
Uric acid	121 (42)	16 (13)	105 (87)	20	88	25	85	1.7	20.3 (14.6–26.3)	32.5 (12.7–55.8)
Bilirubin	169 (58)	8 (5)	161 (95)	17	99	75	82	11.5	19.1 (14.5–24.3)	75.4 (42.7–94.6)
NT-pro-BNP	123 (42)	23 (19)	100 (81)	26	83	26	83	1.5	20.0 (14.3–26.3)	30.4 (14.0–49.0)
HbA1c	135 (47)	25 (19)	110 (82)	25	83	28	81	1.5	20.3 (14.8–26.3)	29.6 (15.1–47.5)
a-tTG IgA	121 (42)	3 (3)	118 (98)	0	97	0	84	0.0	22.3 (17.5–27.4)	10.0 (0.0–62.1)
ANA, anti-DNA, anti-ENA, anti-cardiolipin	118 (41)	22 (19)	96 (81)	16	81	14	83	0.8	22.5 (16.3–29.0)	19.2 (6.1–38.9)
Hepatitis A	106 (37)	1 (0.9)	105 (99)	0	99	0	83	0.0	21.9 (17.3–27.1)	21.1 (0.1–88.6)
Blood screening	115 (40)	1 (1)	114 (99)	0	99	0	84	0.0	22.0 (17.2–27.1)	23.3 (0.0–89.8)
Borrelia	124 (43)	9 (7)	115 (93)	21	95	44	87	4.4	18.9 (13.4–24.5)	55.0 (25.6–80.8)
Epstein Barr virus	110 (38)	2 (2)	108 (98)	0	98	0	84	0.0	22.1 (17.3–27.3)	13.4 (0.0–73.3)
Cytomegalovirus	110 (38)	3 (3)	107 (97)	12	99	67	86	10.9	19.9 (14.7–25.2)	73.4 (26.3–97.1)
P-PSA (only men)	91 (65)	25 (28)	66 (73)	28	73	28	73	1.0	21.9 (15.5–28.8)	27.1 (13.1–44.4)

^a^LR: likelihood ratio (estimation of how much an abnormal test result increases the probability of cancer).

^b^Negative post-test probability (probability of cancer after a normal test result).

^c^Positive post-test probability (probability of cancer after an abnormal test result).

### Accuracy of tests and examinations

The tests in primary care that yielded the highest LRs for cancer were pulmonary X-ray, PEF and calcium (LR 3.5, 3.2 and 2.7, respectively). It should be noted, though, that the total number of true abnormal PEF-results was low (2 cases), and most likely associated with co-morbidity. The probability of cancer after suspected pathology in pulmonary X-ray was estimated to be 49.6% (positive post-test probability) compared to the pre-test probability of 22.1%, while the probability after a normal pulmonary X-ray result was 12.9% (negative post-test probability) (Supplementary Table 1).

**Figure 2.**
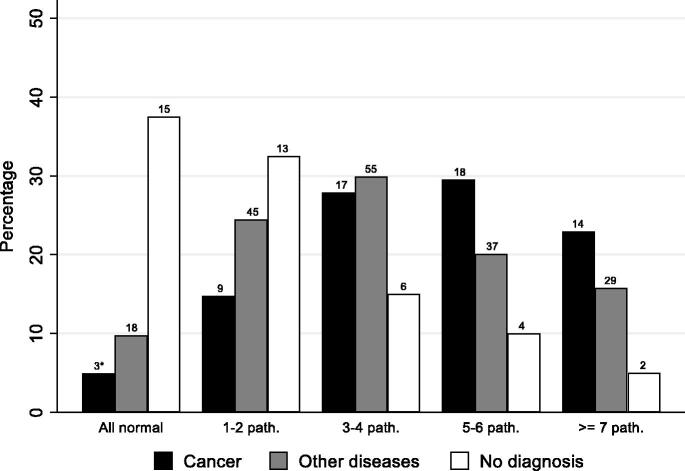
Number of abnormal/pathological test results of compulsory tests (packages 1 + 2 and pulmonary X-ray and ultrasound) in the primary health care. * Number of patients with cancer that had all tests normal.

The number of abnormal test results in primary care differed, as expected, between patients diagnosed with cancer (median 5, IQR 3–6), other diagnoses (median 3, IQR 2–6) and patients who did not get a diagnosis at the DC (median 1, IQR 0–3) (Table 2). In the latter group, 2 (5%) patients had ≥ 7 abnormal test results and 15 (38%) had all normal test results, whereas 14 (22%) of patients later diagnosed with cancer had ≥ 7 abnormal tests and only 3 (5%) had all normal results. Patients diagnosed with other diseases than cancer showed a similar profile as those diagnosed with cancer, although less pronounced (Figure 2). If all tests taken in primary care were normal, this yielded a negative post-test probability of cancer of 12.5% compared to the pre-test probability of 22.1%, while 7 or more abnormal test results increased the post-test probability of cancer to 30.8% (data not shown).

Table 3 shows compliance and test accuracy of the DC package. Bilirubin had the highest LR regarding cancer (LR 11.5) followed by cytomegalovirus (LR 10.9), borrelia (LR 4.4), sodium (LR 3.5) and calcium ion (LR 2.5). The probability of cancer after abnormal bilirubin test was estimated to be 75.4%, while the probability after a negative test was 19.1%. The cancer forms detected after abnormal bilirubin test was hepatic, hematologic and colon cancers. The corresponding post-test probabilities for cytomegalovirus were 73.4% and 19.9%, respectively, and the cancer forms were prostate and hematologic cancers. For both bilirubin and cytomegalovirus, the total numbers of abnormal results were low (8 and 3 respectively). The cancer forms detected after positive borrelia test were lung cancer, prostate cancer, secondary cancer in bone and bone marrow and chronic lymphocytic leukemia (data not shown).

If all tests taken in the DC package were normal, the negative post-test probability of cancer was 11.2% compared to the pre-test probability of 22.1%. However, if 2 tests in the DC package were abnormal, the post-test probability increased to 44.4% (data not shown).

If all recommended physical examinations (inspection) at the DC were deemed normal, the negative post-test probability was 8.9%, while the post-test probability of cancer increased to 31.9% (95% CI 22.0–43.1) with 3 or more abnormal findings (data not shown).

## Discussion

The Swedish DC model started as a pilot project in one region, inspired by the Danish NSSC-CPP. Different variants of the concept are now part of the cancer strategy in Sweden and in all Scandinavia. A previous Swedish evaluation suggested success in view of high patient satisfaction and a cancer detection rate of 22% of the patients, which tallied with the expected proportion at that time [13,15,16]. The present study focused on compliance to, and accuracy of, compulsory tests and examinations. Regarding the test packages 1 and 2, it points towards a low compliance to the workflow in primary care; only 8% of the patients had taken the entire packages. Few single compulsory tests had high accuracy regarding cancer, but a high number of abnormal test results indicated an increased cancer probability.

The study had a prospective design with consecutive inclusion of all patients that were referred to the DC and consented to participate. Data were continuously collected by the DC physician and nurse, which provided consistency, and data were monitored by a research nurse. These are all strengths of the study. However, it has also several limitations. First, the sample size was small considering the heterogeneity of patients with many types of diseases and cancer types. Second, we have only calculated test accuracy regarding cancer, in this case for cancer in general. Many of the compulsory tests were included to identify other diseases and exclude cancer, but we chose to highlight cancer detection; to include all diagnoses would be extremely complex. Another limitation was that we did not have the possibility to collect numerical test results, but only whether the results were abnormal or normal based on reference values. The study should be read with these limitations in mind.

A questionnaire study published in a Swedish journal showed that 94% of primary care physicians thought the DC to be advantageous for patients and 92% thought that it was advantageous for themselves [20]. Nevertheless, compliance to the compulsory test packages in primary care was low, despite large efforts in implementing the model. The main reason for the compulsory test packages was to reduce the diagnostic intervals. The primary care packages were designed by family physicians and the DC package was adapted from the Danish concept by family physicians and specialists in internal and laboratory medicine. During planning of the project, many family physicians were already skeptical to the idea of working with a model with standardized packages since it was contrary to their traditional way of only ordering laboratory tests that were indicated, and there were some virological tests that are usually ordered at the hospitals. The fact that the new concept came from Denmark, which had lower cancer survival rates compared to Sweden [21], also gave rise to hesitation. The low compliance to the laboratory part of the project likely reflects this reluctance. Another reason may be that the catchment area comprised 42 healthcare centers, public and private, with around 300 primary care physicians. To contact all of them and convince them and their managers to implement a new comprehensive work model must have been a considerable challenge (despite financial compensation for the additional tests). In addition, the scientific evaluation includes data from the very first period of the implementation process. Although the model has been described in several studies [13,16,17,22], its superiority, e.g. regarding survival and time saving, compared to usual care has yet to be shown and this may also have affected the attitudes towards the project. Despite the mentioned reluctance to take the entire test packages, this first DC-project in Sweden was generally appreciated, not least by the patients who were offered a fast-track examination [13]. It became a model for DCs all over Sweden. Updated Swedish guidelines regarding diagnostic workup in primary care for patients with nonspecific symptoms still include a compulsory laboratory test package, although less comprehensive than in the present model [23], thus further evaluations of compliance are warranted.

Patients later diagnosed with cancer had fewer compulsory tests taken in primary care than patients with other diseases or no diagnosis. It is possible that experienced family physicians sensed that these patients had, without doubt, serious disease and needed prompt referral without further investigations. A Danish evaluation suggested general practitioners’ gut feeling to be a strong predictor of cancer; an intuitive feeling that the patient was ill despite lack of clinical indications was associated with a cancer probability of 24% [17].

The most common compulsory examination in primary care was diagnostic imaging, and pulmonary X-ray belonged to the examinations with a relatively high test accuracy regarding cancer. Few other single compulsory tests had high accuracy regarding cancer. The diagnostic tests with the highest LRs in primary care were pulmonary X-ray, PEF and plasma calcium, though all with LRs for cancer below 4. In the DC package, bilirubin and cytomegalovirus had LRs for cancer above 10, but it should be noted that for both these tests, the total number of abnormal results was low. Bilirubin is a marker for hepato-pancreato-bilary diseases and for anemia, thus an appropriate test for the studied population. Cytomegalovirus causes a very common infection, which is usually asymptomatic for the young and healthy, but may be pathogenic for elderly and immunosuppressed individuals [24] and is suggested to have oncogenic properties [25]. Interestingly, borrelia had a relatively high LR of cancer too (4.4). High LRs in these cases may partly be due to a low total number of positive tests and a high specificity, which renders a high LR. There are studies that have associated borrelia infection with cancer, more specifically lymphoma [26,27], but evidence is lacking for solid tumors [28]. Sodium and calcium both had LRs above 2. A recent study showed that hyponatremia (serum sodium <135 mmol/L) may be effective as an early marker of many cancer types, including occult cancer [29], and calcium is considered an unspecific cancer marker, supporting their roles in the DC package.

We find it hard to judge which single tests in the test packages that were appropriate based on our results, but there was no doubt that a high number of abnormal test results was more prevalent among patients diagnosed with cancer, especially compared to patients without diagnosis. This was not surprising and confirms findings in studies of the Danish NSSC-CPP [16,17]. If all tests in the DC package were deemed normal, the post-test probability of cancer was halved compared to the pre-test probability, while two abnormal results doubled the probability of cancer.

The thorough physical examination at the DC turned out to be valuable, as expected, with low post-test probability of cancer if no abnormalities were found, whereas three or more abnormal findings increased the post-test probability markedly.

In conclusion, compliance to the workflow in primary care can be considered low. However, 22% of patients referred to the DC turned out to have cancer, suggesting that the family physicians managed well to identify these patients. To define the contents of test packages for detection of all cancers and other serious diseases is a difficult task. An optimal evaluation of test accuracy requires a much larger sample and includes health economic aspects as well as possible impact on related health services. Our small-scale study should be regarded as hypothesis generating, but it shows a clear association between the number of abnormal test results and cancer probability. Thus, overall test package results can provide guidance on the likelihood of cancer in complicated investigations of suspected malignancies.

## Supplementary Material

Supplemental Material
