# Supplementary material for: Diagnostic center for primary care patients with nonspecific symptoms and suspected cancer: compliance to workflow and accuracy of tests and examinations
Source: Scand J Prim Health Care. 2021 May 6;39(2):148–56. doi: 10.1080/02813432.2021.1913892 (PMC8293951; doi:10.1080/02813432.2021.1913892)
Supplement: Supplemental Material [file IPRI_A_1913892_SM9683.docx]

Supplementary Table 1: Compliance to, and test accuracy of, tests in primary care.

| **Test** | **Taken**  **n (%)** | **Abnormal**  **n (%)** | **Normal**  **n (%)** | **Sens**  **%** | **Spec**  **%** | **PPV**  **%** | **NPV**  **%** | **LR^a^** | **Negative post-test probability^b^**  **(95% CI)** | **Positive post-test probability^c^**  **(95% CI)** |
| --- | --- | --- | --- | --- | --- | --- | --- | --- | --- | --- |
| *Pulmonary X-ray and ultrasound:* |  |  |  |  |  |  |  |  |  |  |
| Pulmonary X-ray | 191 (66%) | 47 (25%) | 144 (75%) | 56% | 84% | 49% | 88% | 3.5 | 12.9 (8.5-18.4) | 49.6 (36.4-62.6) |
| Abdominal ultrasound | 165 (57%) | 72 (44%) | 93 (56%) | 63% | 61% | 28% | 87% | 1.6 | 14.8 (8.8-22.2) | 31.0 (22.1-40.6) |
| *Primary care package 1:* |  |  |  |  |  |  |  |  |  |  |
| Hemoglobin (Hb) | 278 (96%) | 126 (45%) | 152 (55%) | 59% | 58% | 28% | 84% | 1.4 | 16.4 (11.1-22.9) | 28.7 (21.6-37.0) |
| Erythrocyte sedimentation rate ESR) | 249 (86%) | 153 (61%) | 96 (39%) | 77% | 42% | 26% | 88% | 1.3 | 13.7 (7.7-21.4) | 27.1 (20.8-34.3) |
| C-reactive protein (CRP) | 250 (86%) | 133 (53%) | 117 (47%) | 64% | 50% | 26% | 84% | 1.3 | 17.0 (11.1-24.2) | 26.6 (19.7-34.3) |
| Glucose | 186 (64%) | 28 (15%) | 158 (85%) | 30% | 88% | 36% | 85% | 2.6 | 18.2 (13.0-23.9) | 42.2 (25.7-59.7) |
| Uric acid | 142 (49%) | 47 (33%) | 95 (67%) | 40% | 68% | 21% | 84% | 1.3 | 19.8 (13.1-27.1) | 26.5 (15.3-39.1) |
| Feces hemoglobin | 115 (40%) | 40 (35%) | 75 (65%) | 33% | 65% | 18% | 81% | 0.9 | 22.4 (15.1-30.2) | 21.3 (11.0-33.8) |
| Electrocardiography (ECG) | 165 (57%) | 32 (19%) | 133 (81%) | 35% | 84% | 28% | 87% | 2.1 | 18.1 (12.4-24.2) | 37.1 (21.8-53.3) |
| Peak expiratory flow (PEF) | 53 (18%) | 8 (15%) | 45 (85%) | 40% | 88% | 25% | 93% | 3.2 | 16.1 (5.9-24.7) | 47.0 (16.0-73.9) |
| Oxygen saturation | 69 (24%) | 2 (3%) | 67 (97%) | 0% | 97% | 0% | 91% | 0.0 | 21.7 (15.4-27.3) | 22.7 (0.1-82.6) |
| *Primary care package 2:* |  |  |  |  |  |  |  |  |  |  |
| Thyroid-stimulating hormone (TSH) | 221 (76%) | 8 (4%) | 213 (96%) | 5% | 97% | 25% | 82% | 1.5 | 21.7 (17.0-26.9) | 30.2 (6.6-63.8) |
| Blood leukocytes | 260 (90%) | 69 (27%) | 191 (74%) | 43% | 78% | 35% | 83% | 1.9 | 17.2 (12.4-22.8) | 35.4 (24.9-46.8) |
| Creatinine | 274 (95%) | 49 (18%) | 225 (82%) | 19% | 82% | 22% | 79% | 1.1 | 21.7 (16.7-27.3) | 23.5 (13.1-36.6) |
| Potassium | 255 (88%) | 16 (6%) | 239 (94%) | 8% | 94% | 25% | 82% | 1.4 | 21.6 (16.8-26.8) | 29.4 (10.8-53.8) |
| Alanine aminotransferase (ALAT) | 255 (88%) | 18 (7%) | 237 (93%) | 8% | 93% | 22% | 79% | 1.1 | 21.8 (17.1-27.0) | 24.0 (8.6-45.8) |
| Gamma-glutamyltransferase (GT) | 236 (81%) | 40 (17%) | 196 (83%) | 23% | 85% | 28% | 82% | 1.5 | 20.4 (15.5-25.9) | 30.2 (17.4-45.4) |
| Alkaline phosphatase (ALP) | 248 (86%) | 48 (19%) | 200 (81%) | 28% | 83% | 29% | 82% | 1.6 | 19.9 (14.9-25.3) | 31.0 (19.1-45.0) |
| Amylase | 176 (61%) | 12 (7%) | 164 (93%) | 9% | 94% | 25% | 82% | 1.4 | 21.4 (16.5-26.7) | 29.7 (9.4-57.3) |
| Calcium | 187 (65%) | 20 (11%) | 167 (89%) | 21.6% | 92% | 40.0% | 82.6% | 2.7 | 19.3 (14.5-24.7) | 43.3 (23.5-64.1) |
| Albumin | 172 (59%) | 53 (31%) | 119 (69%) | 56.7% | 75% | 32.1% | 89.1% | 2.2 | 14.1 (8.7-20.6) | 38.5 (27.1-50.6) |
| Homocysteine | 146 (50%) | 24 (16%) | 122 (84%) | 22.7% | 85% | 20.8% | 86.1% | 1.5 | 20.4 (14.7-26.5) | 29.7 (13.2-49.2) |

^a^LR = likelihood ratio (estimation of how much an abnormal test result increases the probability of cancer)

^b^Negative post-test probability (probability of cancer after a normal test result)

^c^Positive post-test probability (probability of cancer after an abnormal test result)
